# Effect of triple junctions on deformation twinning in a nanostructured Cu–Zn alloy: A statistical study using transmission Kikuchi diffraction

**DOI:** 10.3762/bjnano.7.143

**Published:** 2016-10-24

**Authors:** Silu Liu, Xiaolong Ma, Lingzhen Li, Liwen Zhang, Patrick W Trimby, Xiaozhou Liao, Yusheng Li, Yonghao Zhao, Yuntian Zhu

**Affiliations:** 1 Nano Structural Materials Center, School of Materials Science and Engineering, Nanjing University of Science and Technology, Nanjing, Jiangsu 210094, China; 2 Department of Materials Science and Engineering, North Carolina State University, Raleigh, North Carolina 27695, USA; 3 Australian Centre for Microscopy and Microanalysis, The University of Sydney, Sydney, NSW 2006, Australia; 4 School of Aerospace, Mechanical and Mechatronic Engineering, The University of Sydney, Sydney, NSW 2006, Australia

**Keywords:** nanocrystalline materials, transmission Kikuchi diffraction, triple junctions, twins

## Abstract

Scanning electron microscopy transmission Kikuchi diffraction is able to identify twins in nanocrystalline material, regardless of their crystallographic orientation. In this study, it was employed to characterize deformation twins in Cu/10 wt % Zn processed by high-pressure torsion. It was found that in 83% of grains containing twins, at least one twin intersects with a triple junction. This suggests that triple junctions could have promoted the nucleation of deformation twins. It should be cautioned that this technique might be unable to detect extremely small nanoscale twins thinner than its step size.

## Introduction

The Hall–Petch relationship has inspired materials scientists to refine grains to increase the strength of materials since the early 1950s [1–3]. However, research in this area has met the paradox of strength and ductility tradeoff, especially when materials are refined to ultrafine-grained or nanostructured range [4–8]. They are usually either strong or ductile, but rarely both at the same time. Deformation twinning is one of a few mechanisms that can simultaneously improve both strength and ductility [5–6,9–11]. Consequently, deformation twinning in nanostructured metals has received extensive attention in recent years [10].

Among all of the twinning mechanisms in nanocrystalline materials, partial mission from grain boundaries has been found as the primary mechanism from both simulations [12–13] and experiments [14–15]. However, it still remains unclear whether such emissions from grain boundaries have any preference for sites with specific character such as triple junctions. This problem is of significant importance to both the scientific understanding and practical design of nanostructured materials. Previously, triple junctions have been reported to play a critical role in other deformation mechanisms in nanostructured materials, like grain rotation [16] and grain boundary sliding [17]. Additionally, earlier studies revealed that triple junctions were energetically more active than grain boundaries [18] and they were able to emit and absorb free volumes upon deformation, which promotes partial emission [12]. Therefore, one may reasonably hypothesize that triple junctions may promote deformation twinning in nanocrystalline materials. Up to now, there has been no solid experimental study to verify this issue largely due to technical difficulties.

So far, high-resolution transmission electron microscopy (HRTEM) has achieved great success in revealing the atomic-level details of deformation/growth twins, including formation mechanisms [14,19–22], interactions with dislocations [11,23–26] and macroscopic strain [10,25,27], due to its extraordinary spatial resolution. However, reliable statistical analysis based on extensive data still seems a grand challenge for HRTEM studies on twinning [28–29]. It is time consuming and sometimes impractical to examine twins in a massive number of grains, as each grain needs tilting into a specific zone axis under HRTEM to observe the twin. For example, to observe a twin in a face-centered cubic (fcc) grain, observation along the <110> zone axis on the coherent twin plane is typically needed [29]. In other words, the electron beam needs to be parallel to a close-packed orientation and the twin boundary needs to be edge-on. It is tedious to rotate the sample to various orientations to look for twins in each grain. In addition, the limited sample tilting range makes it impossible to observe all twins in a grain. Therefore, HRTEM substantially underestimates the number of twins in a nanostructured sample. Furthermore, HRTEM also has the same difficulty in characterizing other interfaces like grain boundaries and triple junctions. This is especially problematic when studying the relationship between twins and triple junctions from a statistical view and when the sample has a significant texture.

In the last few years, an innovative microscopy technique has been developed using a conventional electron backscatter diffraction (EBSD) system in a scanning electron microscope (SEM) to collect transmission Kikuchi diffraction (TKD) patterns from sub-10-nm domains in an electron transparent sample [28,30–31]. Like conventional EBSD, TKD can measure grain size, morphology, phase distribution and crystalline lattice orientation. However, the biggest advantage of TKD compared with EBSD is its improvement in spatial resolution, which has been shown to be in the range of 2–10 nm for a variety of materials [30]. Therefore, this technique enables researchers to characterize, with relative ease, truly nanostructured materials. Although strongly dependent on materials and deformation state, the resolution of TKD can complement TEM to provide useful information in characterizing nanostructured materials.

Another great advantage of TKD is that various interfaces, including grain boundaries and twin boundaries, can be identified without the requirement of them being edge-on. Therefore, no tedious sample tilting is required, and all twins can be identified irrespective of their orientation. Furthermore, TKD and subsequent data processing are time/labor-saving and relatively easy due to fast indexing rates and a high level of automation [28,31]. This is in sharp contrast with HRTEM for which the analyses are time consuming and difficult to perform. In addition, comprehensive microstructural information is collected simultaneously from samples analyzed by TKD. This eliminates any subjective judgment of the researcher. Therefore, TKD can achieve the desired balance between spatial resolution, angular accuracy and a high level of automation to give objective results. With these advantages, TKD is ideal for performing statistical analyses on deformation twinning and triple junctions in nanostructured materials.

## Experimental

A 1 mm thick sheet of commercial Cu/10 wt % Zn was first homogenized at 700 °C for 3 h, cooled down to room temperature, and then punched into 10 mm diameter disks for high pressure torsion (HPT) processing. The HPT processing was conducted at room temperature for ten revolutions with an imposed pressure of 1 GPa at a speed of 1.5 rpm. All electron-transparent TKD foils were mechanically ground and punched from the outer region of the as-deformed disks, where the greatest degree of grain refinement was achieved. Samples were subsequently electropolished by means of double-jet electropolishing using an electrolyte of 1:1:2 H_3_PO_4_/C_2_H_5_OH/H_2_O. TKD characterization was conducted in a Zeiss Auriga SEM operating with a 30 kV accelerating voltage. The measurement step size was set to 6 nm for the microscopic observation, taken into consideration the heavily deformed state and other factors. All misindexed points and unindexed pixels in the data sets were screened out prior to analyzing twins. Nearly 1500 grains, ranging in size from 30 to 200 nm, were characterized to enable a statistical analysis of the twinning. TEM observations were performed in a JEOL-2010F microscope under 200 kV as a control group.

## Results and Discussion

In this work, we investigated deformation twinning in a nanostructured Cu–Zn alloy using TKD. Two kinds of twinning phenomena were statistically studied using large TKD data sets: 1) their connections with triple junctions, and 2) the effect of grain size on deformation twinning.

**Figure 1:**
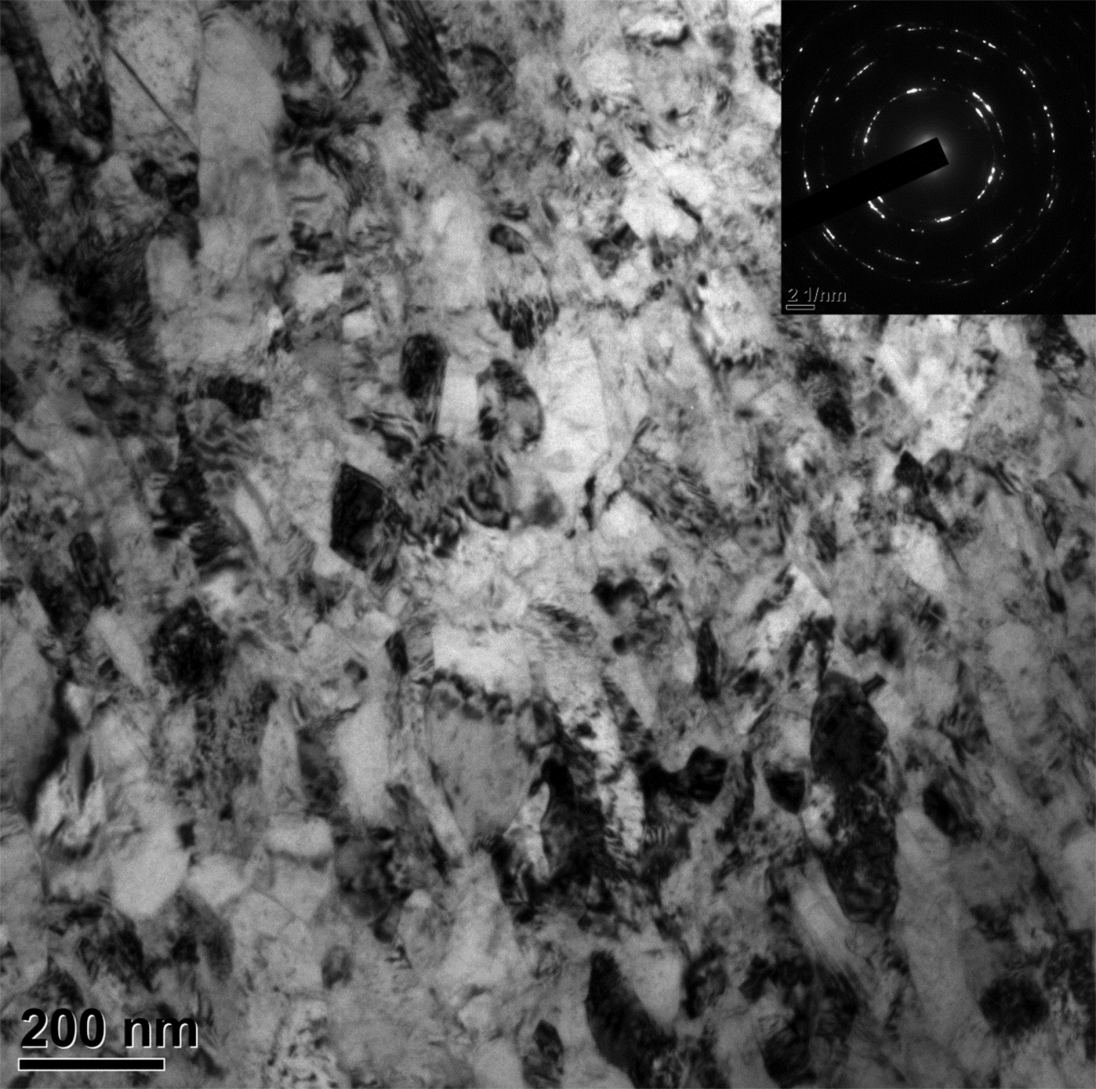
A typical bright-field TEM image of the HPT Cu–Zn alloy showing severe deformation and grain refinement. Inset is a corresponding selected area diffraction pattern.

Figure 1 shows a typical TEM image of the HPT Cu–Zn alloy, revealing equiaxed ultrafine and nanocrystalline grains. Most grain boundaries are not well defined and the interiors of most grains are “messy”, especially in large grains. Those are typical microstructures in highly deformed samples with a high density of entangled dislocations produced by severe plastic deformation [32]. The inset shows a corresponding diffraction pattern. The presence of smeared and ring-like diffraction patterns implies lots of nanoscale grains and their low-angle misorientations. This result is consistent with previous reports of copper alloys processed by high-strain torsion [33–34].

**Figure 2:**
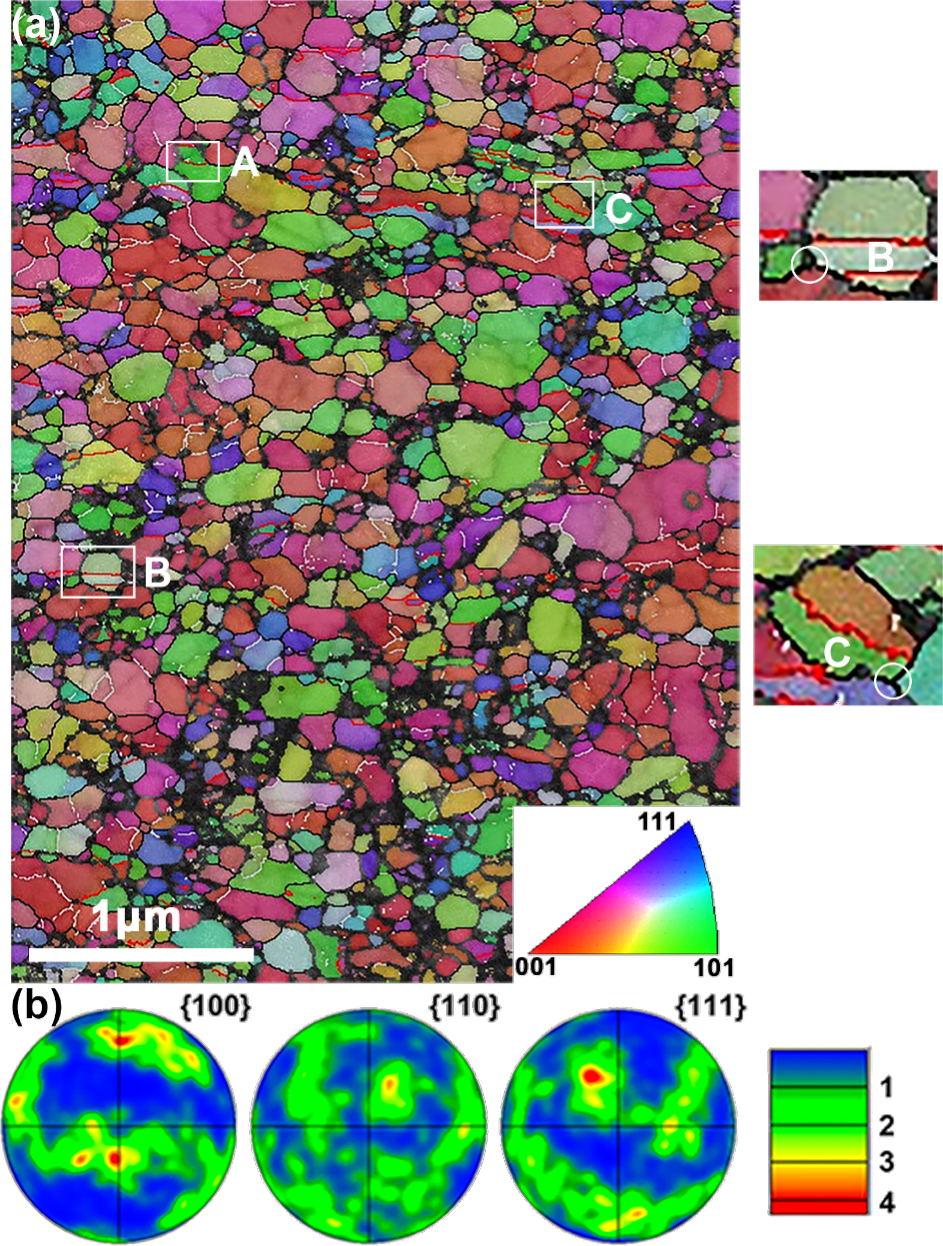
(a) A typical TKD orientation map of the HPT Cu–Zn alloy. The inverse pole figure coloring scheme in the inset is utilized to code the orientation map, in which grains with {001}, {101}, and {111} planes parallel to the sample surface are indicated by red, green and blue, respectively. The black, silver and red boundary lines represent high-angle grain boundaries (>15°), low-angle grain boundaries (2–15°), and Σ3 twin boundaries, respectively. Grains highlighted with white squares and labeled with A, B and C present three typical twin structures that correspond to those shown in Figure 3a–c, respectively. In B and C, twins intersect with triple junctions (highlighted by circles), which is a common phenomenon in all orientation maps. (b) Pole figures of the area presented in (a). Only a weak texture is observed here.

Figure 2a presents a typical TKD orientation map of the HPT-processed Cu–Zn alloy. The orientation map is coded by the inverse pole figure coloring scheme as demonstrated in the inset, in which grains with {001}, {101}, {111} planes parallel to the sample disk surface are indicated by red, green and blue, respectively. The black, silver and red boundary lines correspond to high-angle grain boundaries (>15°), low-angle grain boundaries (2–15°), and Σ3 twin boundaries, respectively. The black points are unindexed points in the orientation map. As shown, the overview of grain size and morphology agrees well with the TEM observation. Severe deformation has induced grain refinement down to the nanoscale. Equiaxed nanoscale grains were found dotted around ultrafine-grains. The grains highlighted by A, B and C have a specific twin feature, which will be discussed in detail in the following section. As in B and C, numerous twins are observed to intersect with triple junctions in the orientation maps. Most grains have significant internal orientation change due to the intragranular deformation that occurred during HPT, as indicated by the abundance of low-angle boundaries and a change in color within grains. The pole figures presented in Figure 2b reveal that there is only a weak texture. These details are consistent with the deformation features observed under TEM.

**Figure 3:**
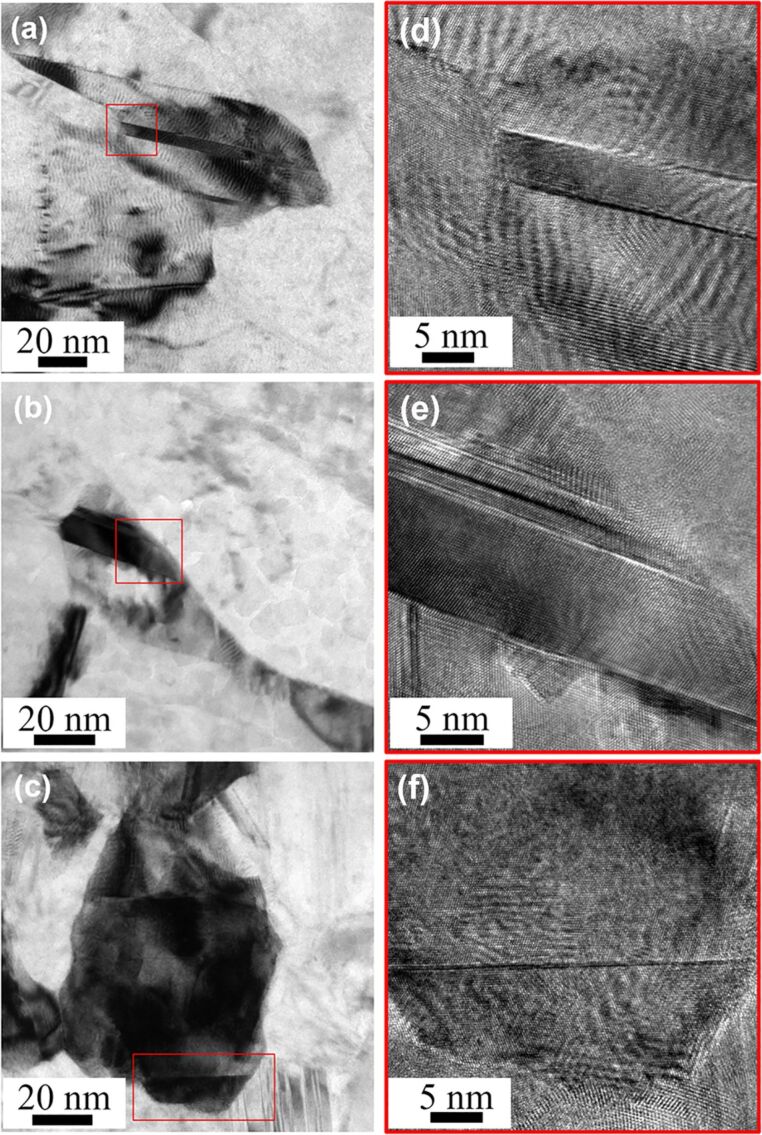
Typical TEM images of the three most common twin morphologies highlighted in Figure 2a: (a) twin thickness is around or slightly larger than the TKD step size; (b) twin thickness is much larger than step size; (c) twin thickness is much larger than step size and there is only one coherent twin boundary in the grain interior; (d), (e) and (f) are enlarged features under HRTEM from (a), (b) and (c), respectively.

Figure 3a–c shows TEM images of three typical types of twins. They corresponds to the three most common twin morphologies observed in TKD orientation maps, as indicated by A, B and C in Figure 2a. These three twins are categorized based on their thicknesses relative to the TKD step size. Generally, for the software to display a twin boundary, it needs to measure the orientation difference across the boundary. But when the twin is extremely small and thinner than one step size (6 nm), it cannot be detected by TKD in our study. Therefore, when the twin domain thickness is around or slightly larger than the step size, as in the case of Figure 3a, TKD is able to detect the twin, but unable to resolve the twin domain. This whole twin appears as one single Σ3 red line in the TKD orientation map and bounded by two separate matrices with the same orientation, as shown in frame A in Figure 2a. Second, when the twin thickness is much larger than the TKD step size (Figure 3b), two Σ3 red lines bounding the twin can be clearly observed, as shown in frame B of Figure 2a. The third case is shown in Figure 3c: when the twinning propagates to the grain boundary and the twin domain thereby becomes large enough to cover part of a grain, only one coherent twin boundary appears in the grain. In this case, a red Σ3 twin boundary line splits the grain with two different orientations, as shown in frame C in Figure 2a.

The statistical information on the relationship of triple junctions and twins is listed in Table 1, which shows results from 300 twinned grains. “Yes” and “No” in the table denote whether a twin intersects with a triple junction. It has been reported that the area nearby a triple junction would emit or absorb free volumes to promote the emission of partial dislocations [12] and then emitted partial dislocations would induce the growth of stacking faults or twins [14–15]. Therefore, a twin meeting the triple junction could be nucleated from the triple junction. The statistics in Table 1 reveals that 248 out of 300 twinned grains (83%) have at least a twin connected with a triple junction. Two grain size subsets, 30–100 nm and 100–200 nm, are selected to make a comparison in order to avoid possible grain size effect on twin–triple junction connections. Each subset has 110 and 190 counted grains, respectively. It is shown that the fractions of twin–triple junction connections are almost the same for the full grain size range, suggesting that there is no discernible grain size effect on twin–triple junction connections. This relatively high percentage of twin–triple junction connections also implies that triple junctions could be playing a significant role in the nucleation of deformation twins. This is the first time such statistical data is reported.

**Table 1:** Statistical data of triple junction related twinning. Yes and No in this table denote whether a twin (boundary or domain) is connected to a triple junction.

Grain size range (nm)	Twinned grain (counts)	No			Yes	
			
		Count	Fraction (%)		Count	Fraction (%)

30–200	300	52	≈17		248	≈83
30–100	110	19	≈17		91	≈83
100–200	190	33	≈17		157	≈83

A well-known deformation mechanism in nanocrystalline fcc metals is partial emission from grain boundaries, resulting in stacking faults and twins. This has been firstly observed through simulation [12–13] and then verified by TEM characterization [14–15]. It has also been demonstrated that triple junctions were energetically more favorable [18,35], and therefore were more effective in promoting partial emissions than grain boundaries. It was found that high densities of triple junctions in nanostructured materials could promote twin nucleation [36]. Although some twins connecting with triple junctions might have not been nucleated at the triple junctions, the statistical results obtained from this investigation suggest that triple junctions might have promoted the nucleation of deformation twins.

Although TKD can only perform post-mortem characterization of nanostructure at this stage, it has the advantage of resolving grain boundaries, triple junctions and twin boundaries without them being edge-on. This makes it more suitable than HRTEM for studying triple junction/twinning relationships. Based on the statistical data, it is reasonable to state that triple junctions in nanostructured metals could play a significant role in the nucleation of deformation twins.

**Figure 4:**
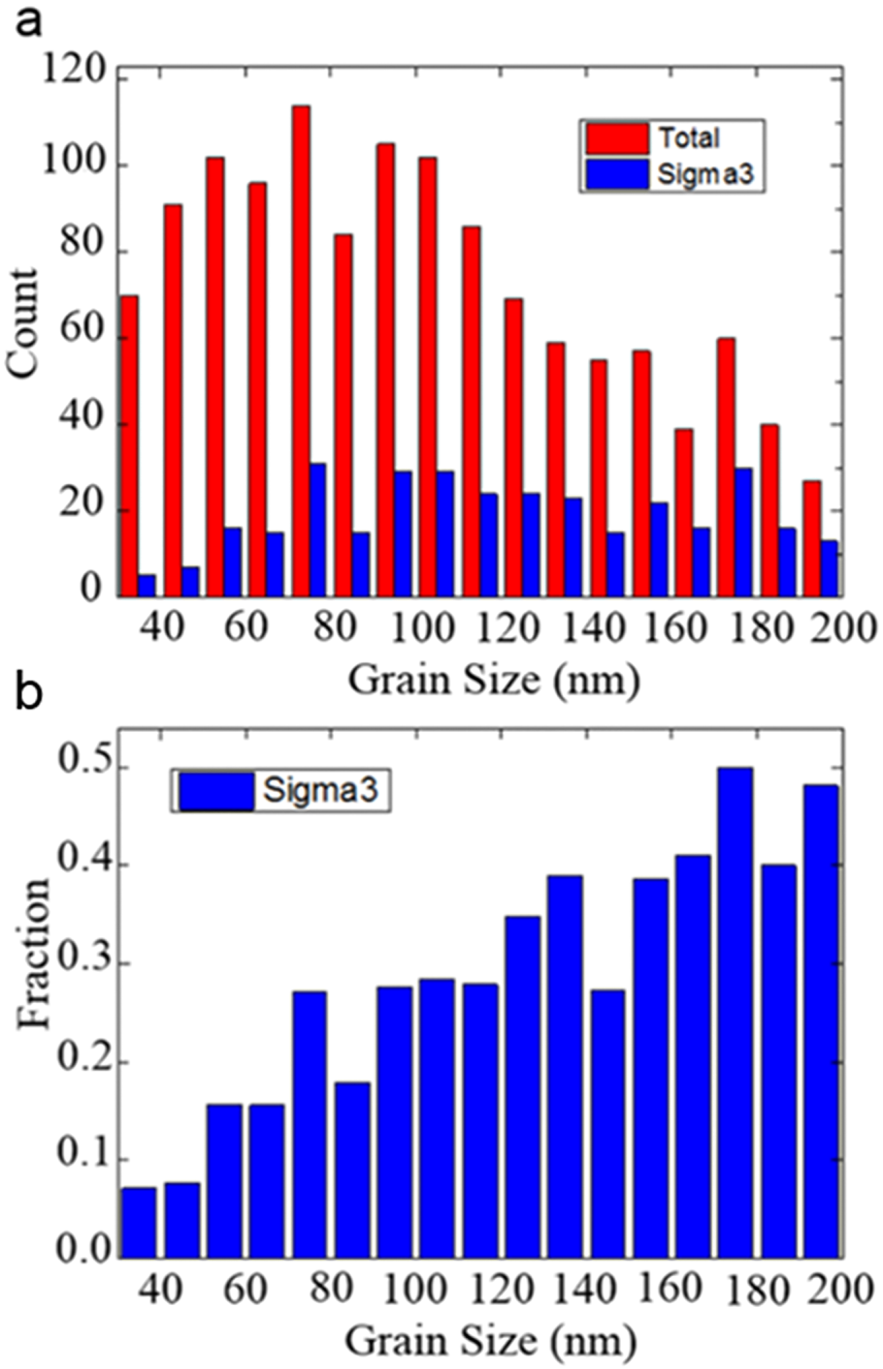
(a) Grain size distribution and the size distribution of twinned grains indicated by red and blue histogram, respectively. (b) Histogram showing the fraction of grains that are twinned in each size range.

Figure 4a shows the grain size distribution and the size distribution of twinned grains in the grain size range from 30 to 200 nm. When analyzing the TKD data, we recorded the information of twinned grains and their sizes. It must be noted that our TKD data volume here is ten times that reported in TEM studies [22,34], and therefore provides more reliable statistics. The average grain size is ≈80 nm. Based on Figure 4a, the fraction of grains that are twinned in each size range is shown in Figure 4b. It clearly reveals an inverse grain size effect, in which the fraction of twinned grains decreases with decreasing grain size [10,22]. However, the optimum grain size for twinning in our observation is not the same as that observed under HRTEM [34]. This discrepancy could be caused by the fact that TKD does not have sufficient resolution to observe very thin twins, that is, twins with a thickness close to or smaller than the 6 nm step size. Smaller grains may contain a large fraction of such small twins, which may have significantly biased the data in Figure 4.

## Conclusion

Compared with HRTEM, TKD has the advantage of easily collecting a large data set for statistical analysis of deformation twinning. In addition, it has the advantage of being able to characterize defect structures, such as grain boundaries, triple junctions and twins, without requiring them to be edge-on and in the <110> zone axis. This makes it possible to detect twins in all orientations. Statistical TKD data reveals that triple junctions could promote nucleation of deformation twins in a nanostructured Cu–Zn alloy. TKD has limitations in detecting thin twins and therefore becomes unreliable when the grain size is thinner than its step size.
